# H-CoNiSe_2_/NC dodecahedral hollow structures for high-performance supercapacitors

**DOI:** 10.1038/s41598-023-29398-y

**Published:** 2023-02-06

**Authors:** P. Salehan, Ali A. Ensafi, Z. Andikaey, B. Rezaei

**Affiliations:** 1 Department of Chemistry, Isfahan University of Technology, Isfahan, 84156-83111 Iran; 2 Department of Chemistry and Biochemistry, University of Arkansas, Fayetteville, AR 72701 USA

**Keywords:** Energy science and technology, Energy storage

## Abstract

The synergistic effect between metal ions and increasing the surface area leads to the fabrication of supercapacitor materials with high capacities. It is predicted that transition metal selenide compounds will be ideal electrode materials for supercapacitors. However, the defects of poor conductivity and volume expansion of the compounds are fundamental problems that must be solved. In this work, we successfully synthesized the cobalt–nickel selenide nitrogen-doped carbon (H-CoNiSe_2_/NC) hollow polyhedral composite structure using ZIF-67 as a precursor. The CoSe_2_ and NiSe_2_ nanoparticles embedded in the NC polyhedral framework offer a wealth of active sites for the whole electrode. Moreover, the presence of the NC structure in the proposed composite can simultaneously lead to improved conductivity and reduce the volume effect created during the cycling procedure. The H-CoNiSe_2_/NC electrode provides high specific capacity (1131 C/g at 1.0 A/g) and outstanding cyclic stability (90.2% retention after 6000 cycles). In addition, the H-CoNiSe_2_/NC//AC hybrid supercapacitor delivers ultrahigh energy density and power density (81.9 Wh/kg at 900 W/kg) and excellent cyclic stability (92.1% of the initial capacitance after 6000 cycles). This study will provide a supercapacitor electrode material with a high specific capacity for energy storage devices.Please confirm the corresponding affiliation for the 'Ali A. Ensafi' author is correctly identified.Error during converting author query response. Please check the eproofing link or feedback pdf for details

## Introduction

Due to the high levels of pollution, the lack of non-renewable energy sources, and the ever-increasing need for energy with the development of human society, it is imperative to find an energy system that is renewable, safe, green, efficient, and affordable^1^. Therefore, the reconnaissance of renewable and efficacious energy sources and the development of energy storage devices have become topics of interest to researchers^2^. Supercapacitors can be applied to portable electronic devices such as flashlights, portable media players, camera flashes, PC cards, automatic meter reading (AMR) systems, and so on^3^. Hence, supercapacitors have wide applications in the field of energy storage^4^. Supercapacitors^5^ are distinguished from traditional capacitors and rechargeable batteries due to their high power density, high charge–discharge rates, and long cycle lives^6^. However, so far, the low energy density of supercapacitors has restricted their practical applications. To improve the energy density in supercapacitors and maintain their intrinsically high power density, it is necessary to improve both the capacitance of the device (C) and the working voltage (V) according to the energy density equation (E = 0.5 CV^2^)^7^. As we know, the intrinsic characteristics of the electrode materials, such as their large specific surface area, high chemical stability, good electronic conductivity, particular microtopography (such as 2D nanosheets and 3D lattices), and presence of two or more oxidation states (for compounds of transition metals), are what determine the capacitance of supercapacitors^8^.

Among the various anode materials that have been perused, transition metal selenides (TMSes) have become advanced anode materials in supercapacitors due to their excellent electrochemical performance^9^. Compared to metal oxides and sulfides, selenides are more appropriate materials due to their favorable electrochemical activity, admirable rotational stability, and greater electrical conductivity^10^. Selenium (Se), which belongs to group VI of the periodic table, generally has better metallic and electrical properties than sulfur (S)^11^. Se has superior electroactivity and more rapid electrochemical reactions because its conductivity (1 × 10^−3^ S m^−1^) is higher than that of S (5 × 10^−28^ S m^−1^)^10^. Therefore, TMSes can act as suitable supercapacitor electrode materials^12^. In addition, bimetallic selenides have more electrochemical redox reactions and better electrochemical activity than monometallic selenides due to their richer redox states^13^. Among many metallic elements, nickel and cobalt are widely used to make positive electrodes for devices due to their acceptable electrical conductivity, desired redox activities, and various valence states^13^.

Metal–organic frameworks (MOFs) are a class of porous materials that are formed by the self-assembly of metal ions and organic ligands^14^. Recently, due to the porosity, favorable electrical conductivity, and rich capacities of MOFs, the use of these compounds in energy storage has received more attention^15^. Zeolite imidazole framework (ZIF), which is a typical MOF, is an appropriate template for the fabrication of hollow structures with a large specific surface area, hierarchical pore size, high electrical conductivity, and redox activity, and its development largely promoted the development of high-performance supercapacitors^16^. The N atom in the imidazole ring is attached to the metal ion to make a tetrahedral structural unit through the coordination point of the divalent middle metal ion (Co^2+^, Zn^2+^, etc.), and then the final 3D framework structure is formed^17^. Since 2-methylimidazole, which is the precursor organic ligand of ZIF, is rich in nitrogen, N-doped porous carbon can be obtained after carbonization, which converts it into a suitable carrier for the preparation of transition metal selenides^18^.

To acquire an attractive structure with an optimal surface area, the rational design and construction of electrode materials are necessary in addition to selecting appropriate materials^19^. Among the variant nanostructures, hollow structures are advantageous for increasing the supercapacitor performance of electrodes due to their vacant interior space and high porosity^20^. In addition, the hollow structure is generally composed of nanoparticles that provide short paths for the penetration of ions and electrons^21^. Therefore, the excellent surface area that provides a good electrode/electrolyte contact area facilitates the electrochemical reaction^22^. On the other hand, the type, morphology, and design of the electrode materials used in supercapacitors significantly affect their structural performance and stability^23–25^. It is acknowledged that it would be advantageous to create hollow nanoporous structures in order to weaken their brittle nature^26^.

Inspired by the above points, in this work, 2-methylimidazole was used as an organic linker and the cobalt ion functioned as the metal node to form single metal crystals of ZIF-67 in a methanol–ethanol solution using the co-precipitation method at room temperature. The previously obtained ZIF-67 was then carbonized for three hours at 650 °C in an argon atmosphere to remove the product from the physically connected group. The content of nitrogen-doped amorphous carbon was then increased and denoted as Co/NC. In addition, we were able to convert the uniform polyhedral microporous Co/NC structure into a hollow porous structure (H-Co/NC) by the etching process with tannic acid. Finally, the obtained product was converted to H-CoNiSe_2_/NC in the presence of nickel salt by the thermal selenization method. Cyclic voltammetry (CV), galvanostatic charge–discharge (GCD), and electrochemical impedance spectroscopy (EIS) were utilized to investigate the electrochemical performance of H-CoNiSe_2_/NC, which was coated on nickel foam electrodes in KOH 3.0 M.

## Results and discussion

### Structural characterization

**Figure 1:**
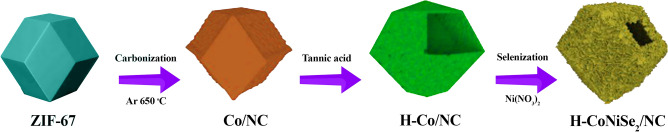
Schematic of H-CoNiSe_2_/NC synthesis.

The synthesis process of H-CoNiSe_2_/NC is schematically shown in Fig. 1. In the first step, single metal ZIF-67 crystals were synthesized as the initial template. In the second step, the synthesized ZIF-67 single metal crystals were carbonized at 650 °C in an argon atmosphere to form Co/NC. In the next step, the uniform microporous, multifaceted Co/NC structure was tuned by the tannic acid etching process and transformed into the hollow porous structure of H-Co/NC. Finally, the H-CoNiSe_2_/NC hollow polyhedral structure was synthesized using a hydrothermal treatment at 200 °C for 12 h.

**Figure 2:**
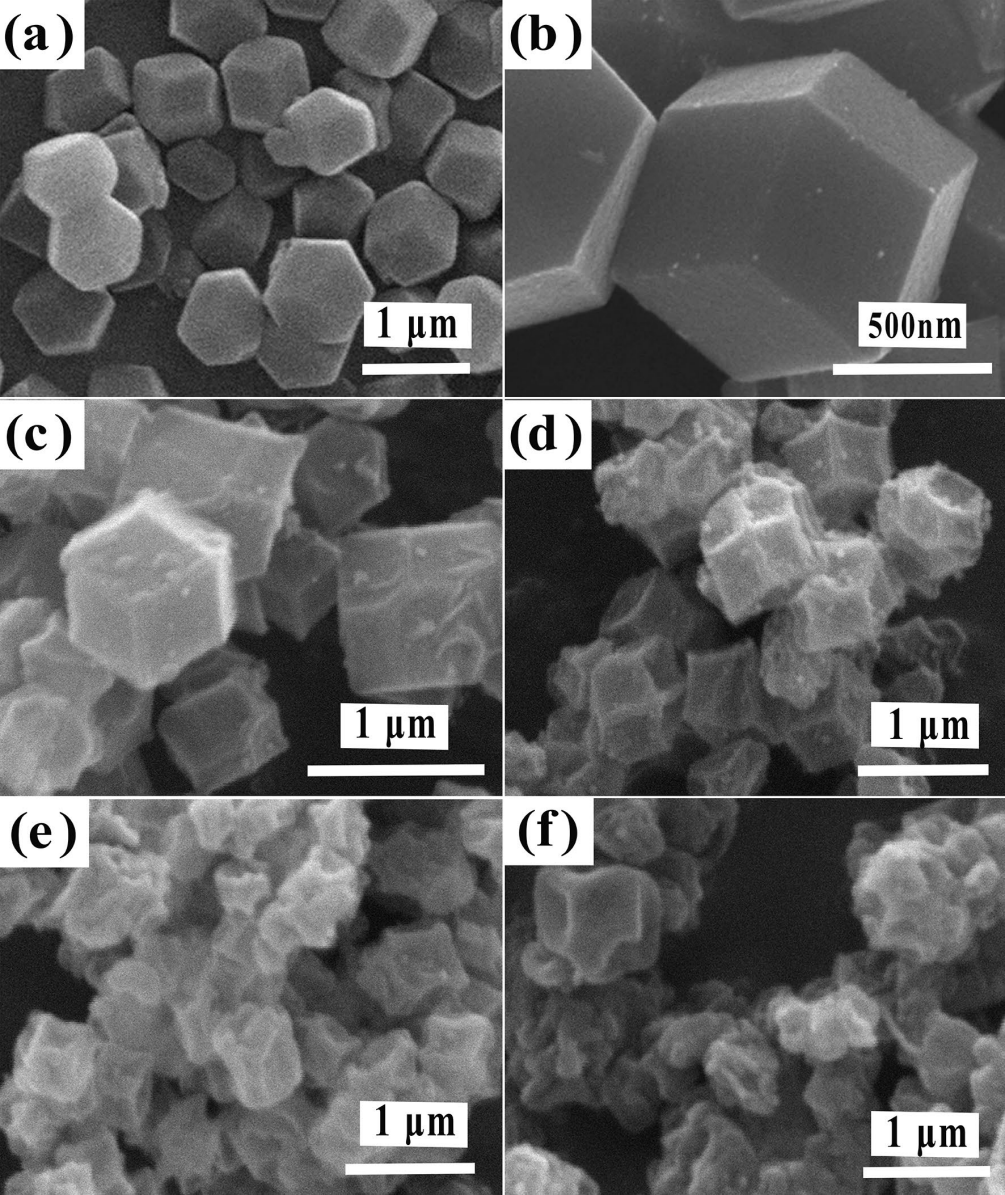
SEM images of (**a** and **b**) ZIF-67, and Co/NC structures at (**c**) 450 °C, (**d**) 550 °C, (**e**) 650 °C, and (**f**) 750 °C.

The recorded SEM images of the various stages of the synthesis of the H-CoNiSe_2_/NC polyhedron derived from ZIF-67 are given to investigate its structure. Figure 2a,b clearly show the formation of regular rhombic dodecahedron morphology with a smooth surface of ZIF-67, which is consistent with previous research. The images of Co/NC at four different temperatures of 450, 550, 650, and 750 °C are shown in Fig. 2c–f. The carbonization temperature affects the structure and surface area of the polyhedrons. As can be seen, with the increase in temperature from 450 to 550 °C, the structure of the polyhedrons has changed; their walls have sunk inward a little and their surface has become rougher, so the increase in their surface area can be seen. At this stage, the carbonization process leads to the transformation of ZIF-67 organic ligands into nitrogen-doped carbon (NC) materials.

**Figure 3:**
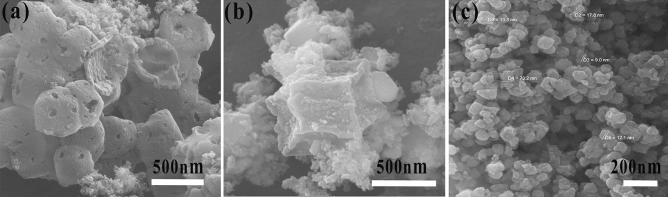
SEM images of (**a**) H-CoNiSe_2_/NC, (**b**) CoNiSe_2_/NC, and (**c**) NiSe_2_ nanoparticles.

The obtained NC particles have retained the ZIF-67's typical polyhedral shape. Compared to ZIF-67, which has a smooth surface, the difference is that the NC particles are slightly shrunk and the surface is roughened^27^. By increasing the temperature to 650 °C, the walls have sunk inward further, so the surface area has again increased. But with a further increase in temperature up to 750 °C, the configuration of polyhedrons is lost, and they have lost their original shape. Therefore, it seems that 650 °C is a more suitable temperature for carbonization. Figure 3a is related to the H-CoNiSe_2_/NC hollow polyhedral structure, which has a hollow structure due to chemical etching with tannic acid. The figure indicates that NiSe_2_ nanoparticles are formed on the polyhedral structure. In addition, the presence of hollow and mesoporous structures is clearly visible. Figure 3b is related to the CoNiSe_2_/NC polyhedral structure without chemical etching with tannic acid. It can be seen that NiSe_2_ nanoparticles are formed on the polyhedral structure, but it is no longer hollow and mesoporous. Figure 3c shows NiSe_2_ nanoparticles, which have particles with an average diameter of 25 nm.

**Figure 4:**
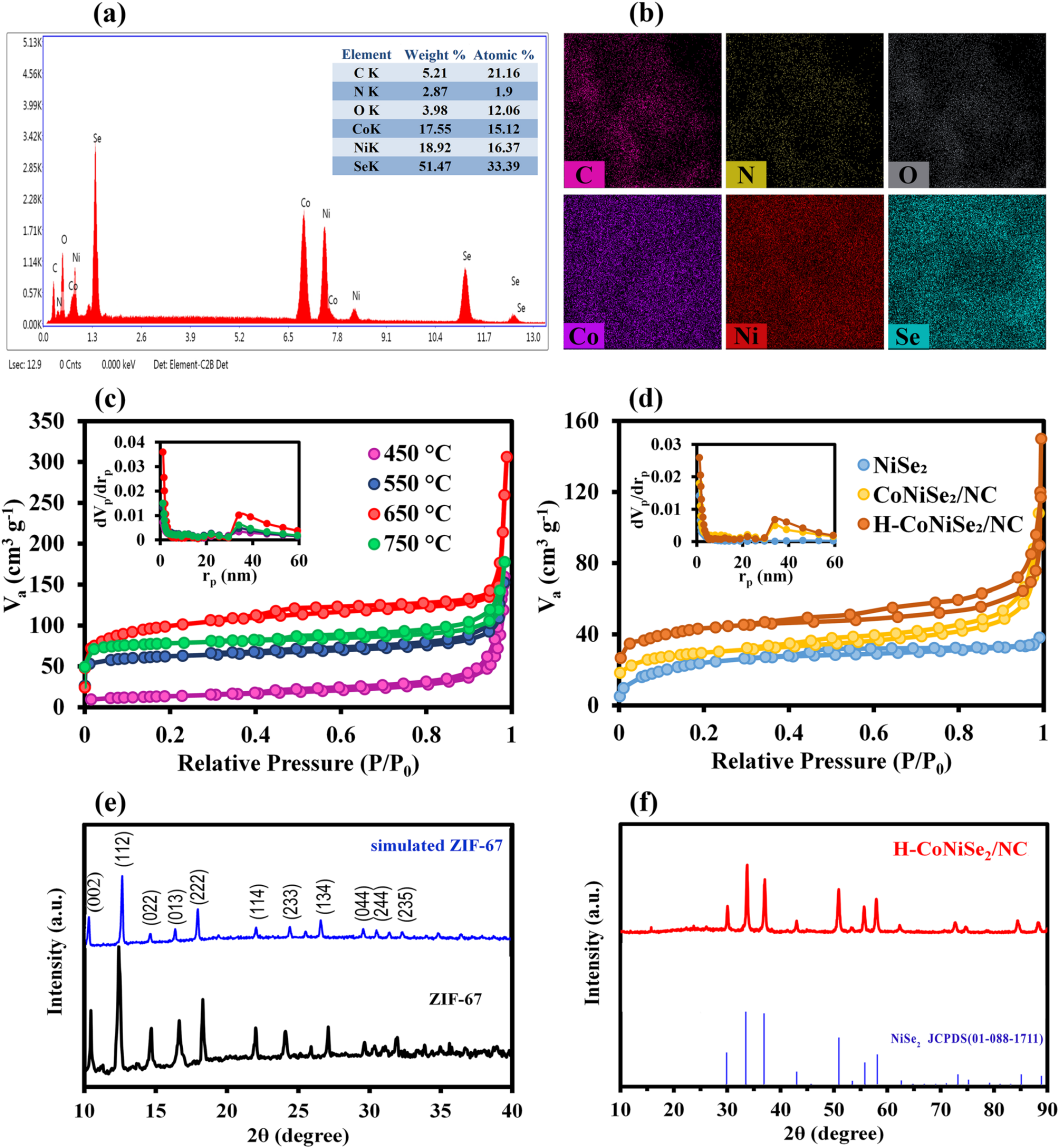
(**a**) EDX spectrum of H-CoNiSe_2_/NC (inset: The weight percentage and atomic percentage analysis of the elements). (**b**) Elemental mapping of C, N, O, Co, Ni, and Se. BET surface area and pore size distribution of (**c**) Co/NC at different temperatures and (**d**) H-CoNiSe_2_/NC, CoNiSe_2_/NC, and NiSe_2_. XRD patterns of (**e**) ZIF-67 and (**f**) H-CoNiSe_2_/NC.

EDX analysis related to the H-CoNiSe_2_/NC hollow polyhedral structure is shown in Fig. 4a. The obtained EDX spectrum confirms the presence of C, N, O, Co, Ni, and Se elements in the composite structure. To better comprehend the contribution of the proposed composite elements, the weight percentage and atomic percentage analysis of the elements was done using EDX, which is inserted in Fig. 4a. Also, the elemental mapping of carbon, nitrogen, oxygen, cobalt, nickel, and selenide in Fig. 4b shows the homogeneous dispersion of the elements in the hollow polyhedral composite.

N_2_ adsorption–desorption isotherms were performed to determine the specific surface area of Co/NC at four temperatures of 450, 550, 650, and 750 °C, H-CoNiSe_2_/NC, CoNiSe_2_/NC, and NiSe_2_. The obtained results from BET analysis in Fig. 4c show that the carbonization temperature affects the specific surface area of ZIF-67 polyhedrons. By increasing the carbonization temperature to 650 °C, the specific surface area increased, but when the temperature increased to 750 °C, the surface area decreased. This reduction of the surface area can be attributed to the change in shape and the destruction of the configuration of ZIF-67 at temperatures above 750 °C. It should be noted that all the samples illustrate a characteristic combination of type I and IV isotherms, which is related to the coexistence of micropores and mesopores. The specific surface area for Co/NC at temperatures of 450, 550, 650, and 750 °C was equal to 52.82, 234.95, 355.41, and 304.82 m^2^/g, respectively. Therefore, 650 °C was chosen as the optimal temperature with the highest specific surface area. As seen in the pore size distribution diagram given in Fig. 4c, two peaks appeared at 1.2 nm and 34 nm, which is a confirmation of micropores and mesopores in the structure of the samples. After chemical etching with tannic acid and selenization of the Co/NC composition, the specific surface area value of the H-CoNiSe_2_/NC decreased to 176.53 m^2^/g (Fig. 4d). This is probably due to partial amorphization and the filling of the pores with NiSe_2_ nanoparticles, which occupy the interior space. The BET surface area of CoNiSe_2_/NC was measured to be 121.47 m^2^/g. The results prove that the H-CoNiSe_2_/NC hollow structure has a higher specific surface area than CoNiSe_2_/NC, indicating that chemical etching increases the electrolyte exposure area. This high surface area can provide available electroactive sites for ion diffusion and transport, thereby improving ion storage efficiency^28^. On the other hand, N_2_ adsorption–desorption isotherms of NiSe_2_ show type I characteristics, demonstrating the existence of micropores. The specific surface area of NiSe_2_ was found to be 75.18 m^2^/g. Also, only one peak at 1.2 nm was observed in the NiSe_2_ pore size distribution diagram, which is in the microporous range. The lower specific surface area and the existence of only micropores in the NiSe_2_ structure lead to fewer active sites and more arduous penetration of electrolyte into the electrode materials during charge–discharge processes.

XRD analysis was utilized to evaluate the crystal structure of the synthesized structures. As seen in Fig. 4e, the spectral pattern of ZIF-67 characteristic diffraction peaks is highly consistent with the simulated XRD pattern of ZIF-67 crystals reported in the literature^29^. This shows that the ZIF-67 polyhedrons have been successfully synthesized and are of high purity. As can be seen, the spectral pattern of H-CoNiSe_2_/NC shown in Fig. 4f is indexed by the NiSe_2_ phase (JCPDS card no. 01-088-1711). No obvious CoSe_2_ phase can be observed, indicating that Co is embedded in the H-CoNiSe_2_/NC lattice.

### Electrochemical performance of manufactured electrodes in a three-electrode system

**Figure 5:**
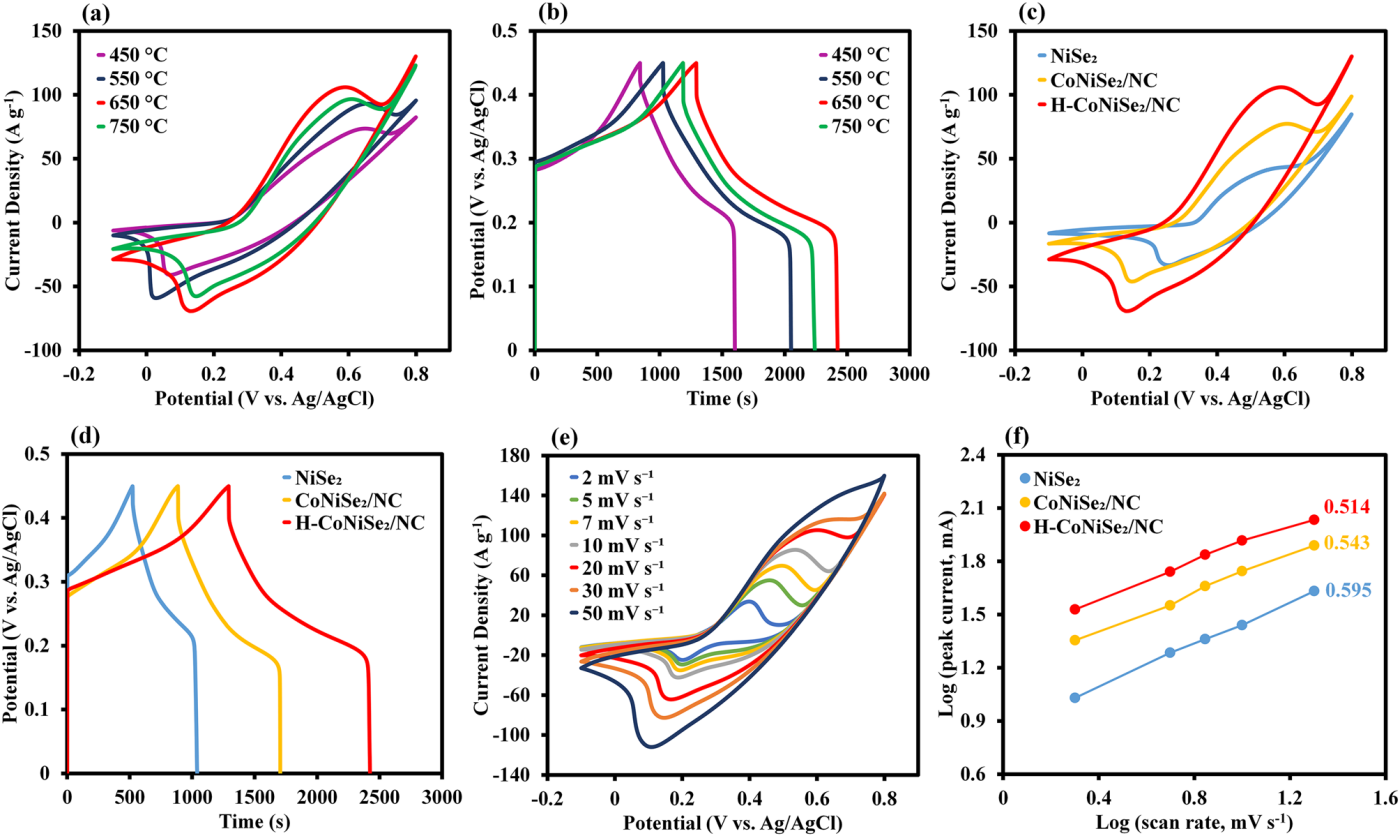
(**a**) CV curves of H-CoNiSe_2_/NC at 20 mV/s at different temperatures. (**b**) GCD curves of H-CoNiSe_2_/NC at 1 A/g at different temperatures. (**c**) CV curves of H-CoNiSe_2_/NC, CoNiSe_2_/NC, and NiSe_2_ at 20 mV/s. (**d**) GCD curves of H-CoNiSe_2_/NC, CoNiSe_2_/NC, and NiSe_2_ at 1 A/g. (**e**) CV curves of H-CoNiSe_2_/NC at various scan rates. (**f**) Log i − Log ν curves of H-CoNiSe_2_/NC, CoNiSe_2_/NC, and NiSe_2_ (cathode scanning).

The electrochemical properties of H-CoNiSe_2_/NC, CoNiSe_2_/NC, and NiSe_2_ as electrode materials for supercapacitors were evaluated using a three-electrode system in a KOH 3.0 M solution. The carbonization temperature of ZIF-67 was optimized to achieve the highest specific capacity. In this way, the CV and GCD curves of H-CoNiSe_2_/NC synthesized with ZIF-67 carbonized at temperatures of 450, 550, 650, and 750 °C were obtained in Fig. 5a,b. The results show that the largest CV enclosed area and discharge time belong to the temperature of 650 °C, which is due to the higher surface area of Co/NC at 650 °C. Therefore, 650 °C was chosen as the optimal carbonization temperature.

For comparison, the CV and GCD curves of H-CoNiSe_2_/NC, CoNiSe_2_/NC, and NiSe_2_ are shown in Fig. 5c,d, respectively. The largest CV enclosed area belongs to H-CoNiSe_2_/NC, which indicates that its high capacity results from the synergistic effect between nickel and cobalt metal ions and abundant active sites. In addition, the discharge time of H-CoNiSe_2_/NC is the longest, indicating that it has the highest specific capacity. The specific capacity values of H-CoNiSe_2_/NC, CoNiSe_2_/NC, and NiSe_2_ were obtained as 1131, 819, and 515 C/g at 1 A/g, which are equivalent to 2262, 1638, and 1030 C/cm^2^ at 2 A/cm^2^, and 22,620, 16,380, and 10,300 C/cm^3^ at 20 A/cm^3^, respectively.

The CV curves of H-CoNiSe_2_/NC at different scan rates from 2 to 50 mV/s in Fig. 5e show a pair of redox peaks that reveal the occurrence of pseudocapacitor-type Faradaic redox Ni^3+/^Ni^2+^ or Co^3+/^Co^2+^ reactions with OH^−^. The suggested redox reactions are as follows^30^:

1 $${\text{CoNiSe}}_{2} + 2{\text{OH}}^{ - } \leftrightarrow {\text{CoSeOH}} + {\text{NiSeOH}} + 2{\text{e}}^{ - }$$

2 $${\text{CoSeOH}} + {\text{OH}}^{ - } \leftrightarrow {\text{CoSeO}} + {\text{H}}_{2} {\text{O}} + {\text{e}}^{ - }$$

3 $${\text{NiSeOH}} + {\text{OH}}^{ - } \leftrightarrow {\text{NiSeO}} + {\text{H}}_{{2}} {\text{O}} + {\text{e}}^{ - }$$

Maintaining the curve shapes at high scan rates indicates fast transport of electrolyte ions to the electrode surface and thus good rate performance. To further investigate the charge storage mechanism of the electrodes, the capacitive contributions of the hybrids were analyzed by CV measurement. Commonly, the relationship between peak currents (i) and the corresponding sweep rates (ν) conforms to the following equations^31^:

4 $$i = a\nu^{b}$$

5 $$log i = b log \nu + log a$$

where b and a are changeable parameters (from 0.5 to 1). If b = 1, the reaction is a surface-controlled (capacitor-type) process, whereas b = 0.5 suggests a diffusion-controlled (pseudocapacitance-type) process^31^. The b values for the cathodic peaks of H-CoNiSe_2_/NC, CoNiSe_2_/NC, and NiSe_2_ electrodes are reflected in Fig. 5f, which are closer to 0.5, demonstrating that the electrodes benefit fr pseudocapacitance-type behavior over capacitive behavior. The H-CoNiSe_2_/NC has a smaller b value than other electrodes, indicating the higher contribution of the diffusion-controlled process. The total capacity was separated into capacitive behavior and diffusion-controlled behavior based on Dunn’s method^32^. Therefore, the current response can be defined as the combination of two separate mechanisms: capacitive contribution (k_1_ν) and diffusion-controlled contribution (k_2_ν^1/2^) at a fixed potential (V). Therefore, we decompose the equation as:

6 $$i\left( V \right) = k_{1} \nu + k_{2} \nu^{1/2}$$

7 $$\frac{i}{{\nu^{1/2} }} = k_{1} \nu^{1/2} + k_{2}$$

In which k_1_ν and k_2_ν^1/2^ represent capacitive and diffusion-controlled processes, respectively. From the linear relationship between i/ν^1/2^ and ν^1/2^, the values of k_1_ and k_2_ can be calculated. It can be noted that the diffusion-controlled process contribution of the H-CoNiSe_2_/NC electrode is 85.6% at 5 mV/s, indicating a diffusion-controlled process.

**Figure 6:**
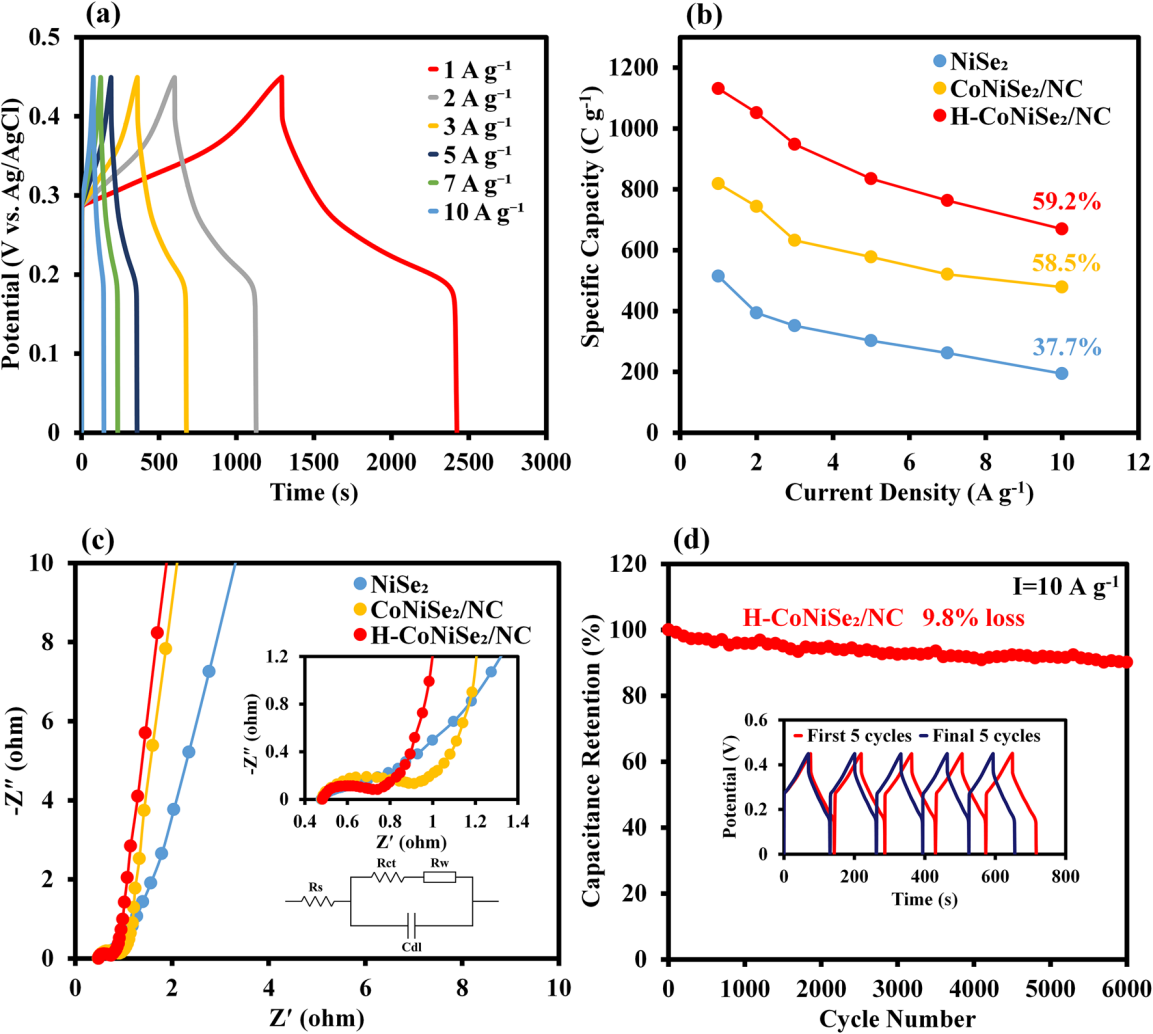
(**a**) GCD curves of H-CoNiSe_2_/NC at various current densities. **(**b) Specific capacities of H-CoNiSe_2_/NC, CoNiSe_2_/NC, and NiSe_2_. (**c**) Nyquist plots of H-CoNiSe_2_/NC, CoNiSe_2_/NC, and NiSe_2_. (**d**) Cycling performance of H-CoNiSe_2_/NC at 10 A/g (inset: GCD curves of the first five cycles and the last five cycles).

The GCD profiles of H-CoNiSe_2_/NC at current densities from 1 to 10 A/g are illustrated in Fig. 6a. The favorable symmetry of the GCD plots at diverse current densities indicates pseudocapacitor behavior and suitable capacitive characteristics due to an ideal reversible redox reaction. The specific capacities of the samples at various current densities are illustrated in Fig. 6b. Compared to NiSe_2_ and CoNiSe_2_/NC, the H-CoNiSe_2_/NC electrode shows higher specific capacities of 1131, 1052, 948, 835, 763, and 670 C/g at current densities of 1, 2, 3, 5, 7, and 10 A/g, respectively, which indicates that H-CoNiSe_2_/NC is an excellent supercapacitor material. It should be noted that with increasing current density, electroactive materials cannot sufficiently participate in redox reactions, which leads to a decrease in specific capacity. In addition, by increasing the current density from 1 to 10 A/g, the NiSe_2_, CoNiSe_2_/NC, and H-CoNiSe_2_/NC electrodes maintain 37.7%, 58.5%, and 59.2% of their initial capacities, respectively. These results show that the H-CoNiSe_2_/NC electrode has a higher rate capability than the CoNiSe_2_/NC and NiSe_2_ electrodes.

The EIS technique can evaluate the charge-transfer resistance (R_ct_) and diffusion of electrolyte ions to the electrode surface^33^. Therefore, EIS spectra were studied in the frequency range of 100 kHz to 100 mHz to further evaluate the transport characteristics of the samples. The corresponding EIS plots with the equivalent circuit model are shown in Fig. 6c. As specified in the model, the R_ct_ value is an important factor in evaluating the electrodes' conductivity and is obtained from the diameter of the semicircle at the high-frequency region. The internal resistance (R_s_) is determined from the intersection of the EIS curve with the Zʹ axis. The slope of the diagonal line at the low-frequency region is related to the Warburg resistance (R_w_), resulting from ions/electrolyte diffusion over electrochemical processes. The impedance spectra in the semicircle region illustrate that the H-CoNiSe_2_/NC electrode has a lower R_ct_ (0.25 Ω) than CoNiSe_2_/NC (0.43 Ω) and NiSe_2_ (0.52 Ω), indicating faster reaction kinetics and remarkable electrical conductivity due to the synergistic effect between Ni and Co ions and the presence of porous carbon in the composite. The steeper slope of the H-CoNiSe_2_/NC electrode compared to other electrodes in the linear region indicates more diffusion of ions to the electrode surface, which the empty spaces in the structure facilitate the transport of ions and electrons.

At 10 A/g, 6000 consecutive cycles were performed to achieve the cyclic stability of the H-CoNiSe_2_/NC electrode. The results in Fig. 6d illustrate that the H-CoNiSe_2_/NC electrode retains 90.2% of its initial specific capacity, indicating relatively excellent cyclic stability. The inserted figure in Fig. 6d relates to the charge and discharge curves of the first five cycles and the last five cycles of the H-CoNiSe_2_/NC electrode. This astounding electrochemical performance of the H-CoNiSe_2_/NC electrode can be attributed to the following:

1. The hollow polyhedral structure is confined with nanoparticles with optimal surface area, which ensures the increase of active sites and the increase of diffusion kinetics.
2. Improved electrochemical stability results from the porous and hollow composite, which reduces structural collapse throughout repeated cycles.
3. Increasing the content of amorphous carbon facilitates the fast transfer of electrolyte ions and increases the rate of the Faraday reaction.
4. Se can significantly improve the conductivity and structural durability of the H-CoNiSe_2_/NC sample^34^.

### Evaluation of the electrochemical behavior of the H-CoNiSe_2_/NC//AC asymmetric supercapacitor

**Figure 7:**
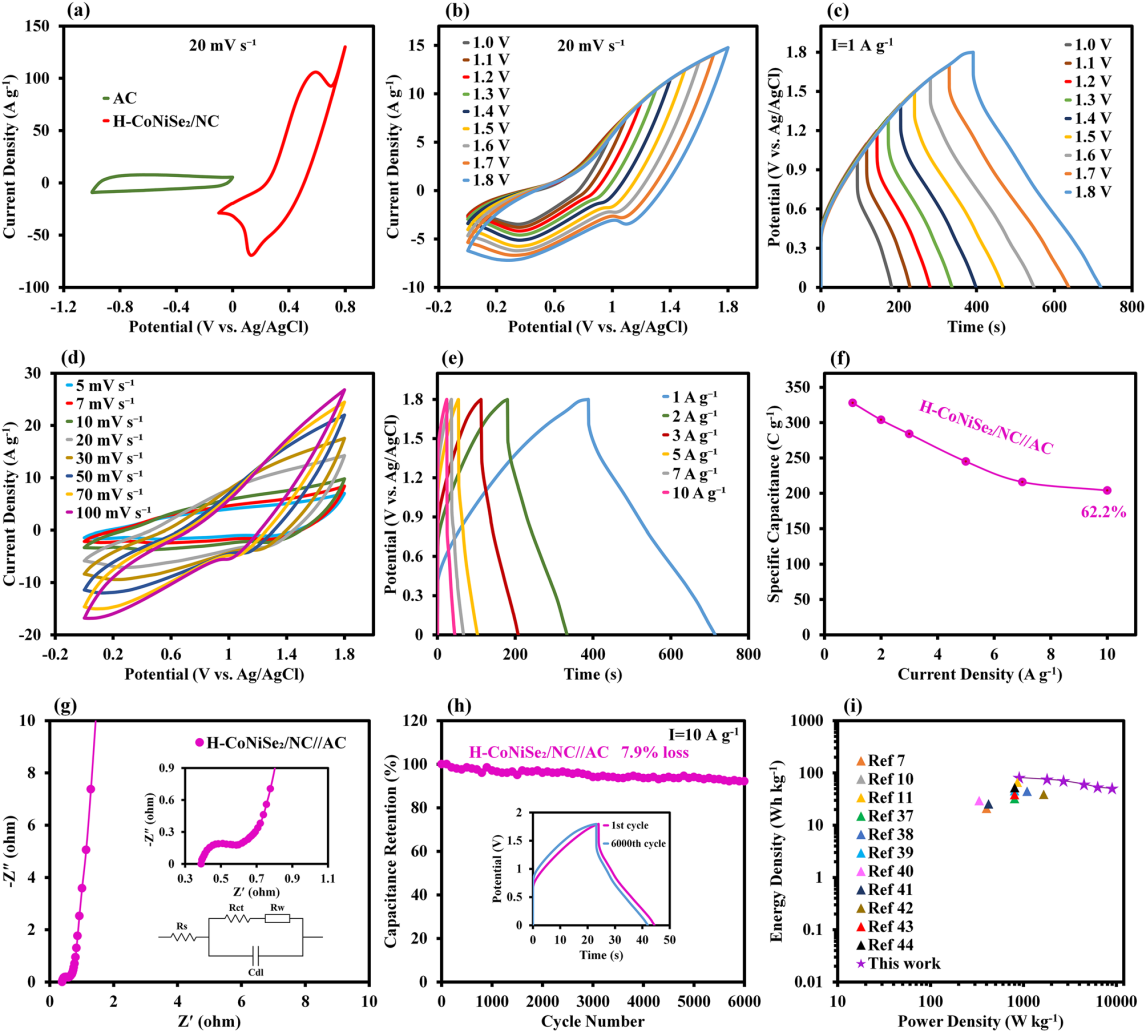
(**a**) CV curves of AC and H-CoNiSe_2_/NC at 20 mV/s in a three-electrode system. (**b**) CV curves of H-CoNiSe_2_/NC//AC at 20 mV/s at various potential windows. (**c**) GCD curves of H-CoNiSe_2_/NC//AC at 1 A/g at various potential windows. (**d**) CV curves of H-CoNiSe_2_/NC//AC at various scan rates. (**e**) GCD curves of H-CoNiSe_2_/NC//AC at various current densities. (f**)** Specific capacitances of H-CoNiSe_2_/NC//AC at 1 to 10 A/g. (g) Nyquist plot of H-CoNiSe_2_/NC//AC. (**h**) Cycling performance of H-CoNiSe_2_/NC//AC at 10 A/g (inset: GCD curves of the first and last cycles). (**i**) Ragone plot of H-CoNiSe_2_/NC//AC.

To investigate the practical application of H-CoNiSe_2_/NC, the corresponding asymmetric supercapacitor (ASC) is assembled by using H-CoNiSe_2_/NC as the positive electrode, AC as the negative electrode, and potassium hydroxide as the electrolyte (defined as H-CoNiSe_2_/NC//AC). Since the anode and cathode electrodes have different potential windows, using an asymmetric arrangement leads to an expansion in the operating voltage window and improved energy density^35^. To determine the operating potential window of the H-CoNiSe_2_/NC//AC asymmetric supercapacitor, the CV curves of H-CoNiSe_2_/NC (− 0.1 to 0.8 V) and AC (− 1.0 to 0 V) are obtained at a scan rate of 20 mV/s in a three-electrode system. The appropriate voltage window for the H-CoNiSe_2_/NC//AC device is predicted to be 1.8 V based on the CV curves in Fig. 7a.

To further investigate the potential window range, CV and GCD diagrams are drawn for the H-CoNiSe_2_/NC//AC in different potential windows (Fig. 7b,c). As can be seen, the high voltage range of 1.80 V is suitable for the ASC. In addition, pseudocapacitor behavior at high potentials and EDLC behavior at low potentials are present in the fabricated device, which indicates a good assembly of the hybrid device. The CV curves of H-CoNiSe_2_/NC//AC at different scan rates from 5 to 100 mV/s are shown in Fig. 7d. At all scan rates, both pseudocapacitances from the H-CoNiSe_2_/NC and EDLCs from the AC contribute to charge storage. In addition, the lack of noticeable change in the shape of the CV curves with increasing scan rate indicates the satisfactory rate capability of the device.

Figure 7e depicts the GCD profiles of H-CoNiSe_2_/NC//AC at current densities ranging from 1 to 10 A/g. The GCD curves of the ASC are symmetric at the high voltage window of 1.80 V, indicating the device’s outstanding electrochemical reversibility and favorable mass balancing. The capacitance of H-CoNiSe_2_/NC//AC was obtained from the charge–discharge diagram. In Fig. 7f, the ASC has a good capacitance of 327 C/g at 1 A/g (equivalent to 1635 C/cm^2^ at 5 A/cm^2^, and 16,350 C/cm^3^ at 50 A/cm^3^) and a rate capability of 62% at 10 A/g.

The EIS spectrum of H-CoNiSe_2_/NC//AC was studied in the frequency range of 100 kHz to 100 mHz. The obtained EIS plot along with the equivalent circuit model is shown in Fig. 7g. The R_ct_ value for the ASC was found to be 0.18 Ω, indicating favorable electrical conductivity. To evaluate the cyclic stability of the H-CoNiSe_2_/NC//AC device, 6000 consecutive cycles in 10 A/g are recorded in Fig. 7h, showing that the ASC has great cyclic stability by maintaining 92.1% of the initial capacitance. The Ragone curve of H-CoNiSe_2_/NC//AC, to investigate the energy-storage ability of the ASC, is shown in Fig. 7i. According to the results, the H-CoNiSe_2_/NC//AC device achieves a high energy density of 81.9 Wh/kg at a power density of 900 W/kg (equivalent to 0.409 Wh/cm^2^ at 4.5 W/cm^2^, and 4.088 Wh/cm^3^ at 45 W/cm^3^). Additionally, the ASC still displays an energy density of 50.8 Wh/kg at a higher power density of 9000 W/kg (equivalent to 0.255 Wh/cm^2^ at 45 W/cm^2^, and 2.551 Wh/cm^3^ at 450 W/cm^3^). As seen in Fig. 7i, our ASC has better electrochemical performance than previous reports, which are compared in detail in Table 1.

**Table 1:** Comparison of the proposed electrode with some transition metal selenide electrodes.

Electrode	Capacity (three-electrode system)	Capacitance (two-electrode system)	Energy density (Wh/kg)	Power density (W/kg)	Ref
Co_0.85_Se	294 F/g at 0.5 A/g	–	21.1	400	^7^
CoSe_2_/MoSe_2_	211.97 mAh/g at 1 A/g	64.8 mAh/g at 1 A/g	51.84	799.2	^10^
MNSe@NF	1172.16 C/g at 2 A/g	280 C/g at 1 A/g	66.1	858.45	^11^
CCSe	562 C/g at 2 A/g	144 C/g at 1 A/g	32.4	800	^37^
Co–Mo–Se	221.7 mAh/g at 1 A/g	57.7 mAh/g at 1 A/g	44.7	1094	^38^
NiFe_2_Se_4_	372.2 mAh/g at 1 A/g	126.9 F/g at 1 A/g	45.6	800	^39^
CuCo–Se	503 C/g at 10 mA/cm^2^	132 C/g at 10 mA/cm^2^	29.5	332.9	^40^
Ni_0.85_Se@MoSe_2_	774 F/g at 1 A/g	–	25.5	420	^41^
(Ni,Co)Se_2_/NiCo-LDH	1224 F/g at 2 A/g	102 F/g at 2 A/g	39	1650	^42^
NiSe_2_/CoSe_2_	1668 F/g at 1 A/g	108.2 F/g at 1 A/g	38.5	802.1	^43^
(Ni,Co)Se_2_@rGO	649.1 C/g at 1 A/g	239.8 C/g at 1 A/g	52.6	803.4	^44^
H-CoNiSe_2_/NC	1131 C/g at 1 A/g	327 C/g at 1 A/g	81.9	900	This work

**Figure 8:**
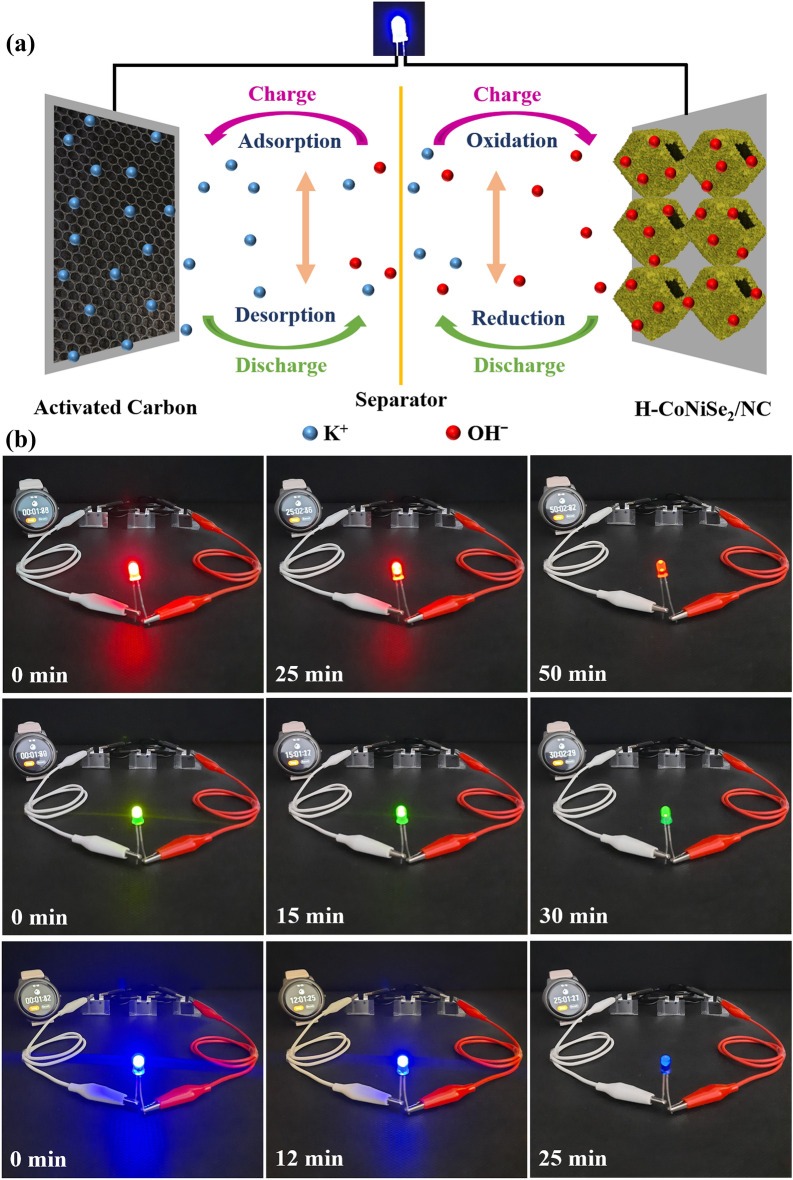
(**a**) Schematic illustration of the H-CoNiSe_2_/NC//AC asymmetric supercapacitor. (**b**) Photographs of red, green, and blue LEDs powered by three H-CoNiSe_2_/NC//AC in series.

The schematic illustration of the H-CoNiSe_2_/NC//AC asymmetric supercapacitor is drawn in Fig. 8a. To demonstrate the actual use, three H-CoNiSe_2_/NC//AC connected in series turned on a red, green, and blue LED individually for 50, 30, and 25 min, respectively (Fig. 8b).

## Methods

### Materials

The chemicals were used without further purification, including 2-methyl imidazole, Co(NO_3_)_2_·6H_2_O, Ni(NO_3_)_2_·6H_2_O, selenium powder, activated carbon, carbon black, methanol, and absolute ethanol, which were purchased from Sigma-Aldrich. Tannic acid (TA), hydrazine hydrate, *N*-methyl-2-pyrrolidone (NMP), polyvinylidene difluoride (PVDF), and potassium hydroxide were purchased from Merck Company. Distilled water was used during all experiments.

### Synthesis of cobalt nitrogen-doped carbon hollow polyhedrons (H-Co/NC)

ZIF-67 polyhedrons were synthesized according to previously reported methods^36^. Typically, 2.183 g of Co(NO_3_)_2_.6H_2_O was added to a mixture of 20 mL methanol and 20 mL ethanol to create solution A, and 1.322 g of 2-methylimidazole was added to a mixture of 20 mL methanol and 20 mL ethanol to create solution B. Subsequently, solution B was quickly added into solution A and stirred vigorously for 30 min. Then, after staying at room temperature for 24 h without stirring, the reaction was carried out, and the obtained ZIF-67 violet precipitate was centrifuged and washed three times with a mixture of methanol and ethanol.

The prepared ZIF-67 was then carbonized at several temperatures (450, 550, 650, and 750 °C) in a tube furnace under an argon atmosphere for 3 h at a ramping rate of 2 °C min^−1^ to form nitrogen-doped carbon containing cobalt particles (Co/NC). Next, 0.1 g of the Co/NC was dispersed in 2 mL of ethanol under ultrasonic and intense stirring to obtain solution A, while solution B was obtained by dissolving 0.25 g of tannic acid in 48 mL of distilled water. Solution B was slowly added into solution A under intense stirring and aged for 10 min to form a hollow structure denoted as H-Co/NC. Then, the H-Co/NC polyhedrons were centrifuged and washed several times with a 50% v/v methanol aqueous solution.

### Synthesis of cobalt–nickel selenide nitrogen-doped carbon hollow polyhedrons (H-CoNiSe_2_/NC)

First, 5 mL of hydrazine monohydrate (N_2_H_4_.H_2_O) was used to dissolve 0.158 g of Se powder to obtain solution A. Then, 0.291 g of Ni(NO_3_)_2_.6H_2_O was dispersed in a mixture consisting of 10 mL of distilled water and 15 mL of ethanol under intense stirring to obtain solution B. At room temperature, solution B was then dropwise injected with solution A, and 0.050 g of HCo/NC was added under vigorous ultrasonication for 2 h. The mixture was then poured into a 50-mL autoclave lined with Teflon and subjected to hydrothermal treatment for 12 h at 200 °C. After cooling, the black product was centrifuged and washed six times with distilled water and absolute ethanol to get H-CoNiSe_2_/NC. For comparison, CoNiSe_2_/NC was synthesized by the same method using Co/NC instead of H-Co/NC. Also, NiSe_2_ nanoparticles without adding H-Co/NC were synthesized using the same procedure.

### Identification equipment

Field-emission scanning electron microscopy (FESEM, TESCAN MIRA3) coupled with energy-dispersive X-ray spectroscopy (EDX) was used to characterize the morphology and elemental composition of samples. X-ray diffraction (XRD, Bruker D8 ADVANCE) patterns were achieved to analyze the crystalline phase of the synthesized samples. The Brunauer–Emmett–Teller (BET) surface areas and pore size distribution were determined from nitrogen adsorption/desorption isotherms by BELSORP-miniX at 77 K.

### Electrochemical evaluation

Electrochemical tests of the prepared electrodes were done by a Bio-Logic SAS potentiostat (SP-300, France). CV, EIS, and GCD techniques were performed to investigate the supercapacitor behavior of the fabricated electrodes in a three-electrode configuration, where the working electrode was coated with a mixture of electroactive material (80%), carbon black (15%), and PVDF (5%), in a few drops of NMP solvent on a nickel foam electrode (1.0 × 0.5 × 0.1 cm), and a platinum rod and an Ag/AgCl foil were used as counter and reference electrodes, respectively. The nickel foam, which was covered with an active electrode material, was dried for 24 h in an oven at 60 °C. The mass of the coated active substance was about 2.0 mg/cm^2^. All experiments were performed in an aqueous medium of KOH 3.0 M. The capacity of our electrodes in the three-electrode system was determined through the GCD diagram using Eq. (8)^34^:

8 $$C_{sp} = \frac{I \times \Delta t}{m}\left( {C\,{\text{g}}^{ - 1} } \right)$$

where C_sp_, I, ∆t, and m are specific capacity (C/g), discharge current (A), discharge time (s), and mass of active material coated on the electrode (g), respectively.

### Fabrication of the asymmetric supercapacitor device

To make an asymmetric supercapacitor (hybrid supercapacitor) device, activated carbon and H-CoNiSe_2_/NC were used as negative and positive electrodes, respectively, in a KOH 3.0 M electrolyte. The mass ratio of positive and negative electrodes was found to be about 0.25 based on the charge equilibrium theory (m+/m^−^) to obtain optimal performance. In the charge equilibrium theory Eqs. (9, 10) given below, Q, C, m, and ΔV are stored charge (C), capacity (F/g), the mass of electrode material (g), and potential range (V), respectively^22^.

9 $$Q = C \times \Delta V \times m$$

10 $$\frac{{m^{ + } }}{{m^{ - } }} = \frac{{C^{ - } \times \Delta V^{ - } }}{{C^{ + } \times \Delta V^{ + } }}$$

The specific capacitance of the asymmetric device was obtained from the GCD curve according to the following equation:

11 $$C_{s} = \frac{I \times t}{m}\left( {C\,{\text{g}}^{ - 1} } \right)$$

where the symbols are related to specific capacitance (C_s_, C/g), discharge current (I, A), discharge time (t, s) , and total mass placed on the both electrodes (m, g)^37^.

Energy density (E) and power density (P) are two key supercapacitor parameters, represented by the following equations^22^:

12 $$E = \frac{{C \times \Delta V^{2} }}{7.2}$$

13 $$P = \frac{3600 \times E}{{\Delta t}}$$

Here, E, P, C, ∆V, and t are specific energy (Wh/kg), specific power (W/kg), capacitance (F/g), potential window (V), and discharge time (s), respectively.

## Conclusion

In conclusion, a hollow polyhedral construction prepared via nickel selenide nanoparticles embedded in H-Co/NC (H-CoNiSe_2_/NC) was developed. Taking advantage of the logical architecture between the hollow polyhedral structures of the H-Co/NC and NiSe2 nanoparticles leads to consistent electrochemical properties in the composite of the H-CoNiSe_2_/NC. This unique hollow and porous polyhedral structure leads to abundant active sites and rapid electrolyte diffusion to access internal and external surfaces. In addition, the decorated carbon in the modified structure with metal selenides can facilitate conductivity during the electrochemical process. The as-fabricated H-CoNiSe_2_/NC electrodes exhibited a high specific capacity of 1131 C/g at 1.0 A/g, accompanied by a rate capability of 59.2% retention of the initial capacity at 10 A/g. Additionally, 90.2% of the initial capacity was maintained after 6000 cycles at a current density of 10 A/g. The corresponding H-CoNiSe_2_/NC//AC hybrid supercapacitors delivered an ultrahigh energy density of 81.9 Wh/kg at a power density of 900 W/kg with a high cyclic stability of 92.1% of the initial capacity after 6000 cycles at a current density of 10 A/g. This investigation confirms that H-CoNiSe_2_/NC can act as a high-performance supercapacitor.

## Data Availability

The datasets used and/or analyzed during the current study are available from the corresponding author on reasonable request.
